# Supplementary material for: Cation-induced changes in the inner- and outer-sphere mechanisms of electrocatalytic CO2 reduction
Source: Nat Commun. 2023 Nov 22;14:7607. doi: 10.1038/s41467-023-43300-4 (PMC10665450; doi:10.1038/s41467-023-43300-4)
Supplement: Supplementary file 1 — Supplementary Information [file 41467_2023_43300_MOESM1_ESM.pdf]

Supporting Information for
Cation-induced changes in the inner- and outer-sphere mechanisms of electrocatalytic CO_2 reduction

Xueping Qin[1*], Heine A. Hansen[1], Karoliina Honkala[2] and Marko M. Melander[2*]

[1]Department of Energy Conversion and Storage, Technical University of Denmark, Anker Engelunds Vej Building 301, Kgs. Lyngby, 2800, Denmark.
[2]Department of Chemistry, Nanoscience Center, University of Jyväskylä, P.O. Box 35, FI-40014, Jyväskylä, Finland.

*Corresponding author(s). E-mail(s): xueqi@dtu.dk; marko.m.melander@jyu.fi;

S.1 OS-ET analysis

S.1.1. Au-water-2Li

For the Au-water-2Li interface, the cDFT-MD simulations are performed. The energy gap distributions are sampled and key structures of diabatic states of OS-ET at Au-water-2Li interfaces are shown in Fig. S1. With the presence of Li cations, the reorganization energy is calculated to be 7.60 eV with a reaction energy of 3.63 eV. The Marcus energy barrier (4.15 eV) is even higher than the Au-water-2K system (2.93 eV). Such a difference can be ascribed to the different solvation properties of Li and K: Li is strongly hydrated compared to K and has a higher solvation energy.[1] This indicates that the interactions between Li cations and surrounding water molecules are stronger than K-H_2O, leading to a higher reorganization energy and thus a high Marcus barrier.

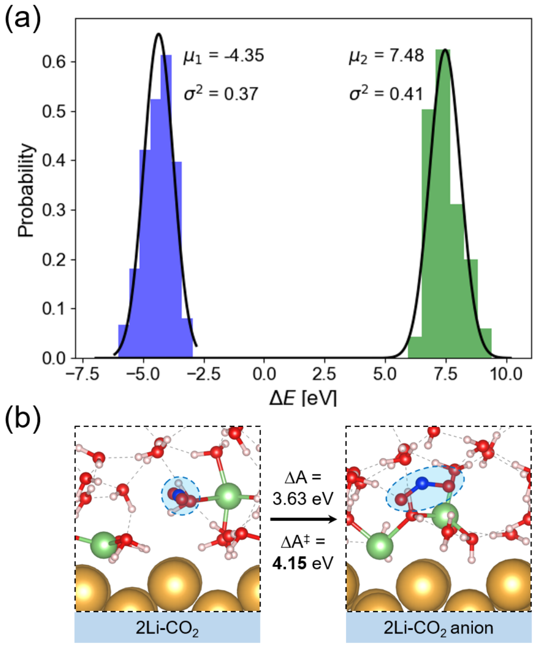

Fig. S1: The energy gap distributions (a) and key structures of diabatic states (b) of OS-ET at Au-water-2Li interfaces.

S.1.2. Constant electrode potential corrections and Fermi levels for interfacial models

We have estimated the impact of constant potential corrections within the capacitor model.[2] The resulting energy corrections are quite small as shown in Table S1. For the 2K system, the Fermi level difference between the initial (2K-CO_2) and final states (2K-CO_2 anion) is only 0.28 eV; even if we consider the maximum charge difference of 1e, the energy correction within the capacitor model is only 0.14 eV. For the water system, the Fermi level difference is even smaller (0.08 eV) with a negligible energy correction of 0.04 eV. Comparing the water-CO_2 and 2K-CO_2, the Fermi level difference is also very small, 0.28 eV; water-CO_2 anion and 2K-CO_2 anion show the Fermi level difference of 0.08 eV.

Adding cations to the solution with neutral CO_2 should make the surface more negative, which is observed. In the water case, the Fermi level is changed only by 0.08 with a variance of 0.25 comparing the water-CO_2 and water-CO_2 anion systems, meaning that it is basically the same. It is also possible that the Fermi level of the water-CO_2^- system is determined by CO_2^- rather than the surface, which defines the Fermi level in the water-CO_2 system. The analysis of the density of states (DOS) and projected density of states (PDOS) supports this assertion as there is a clear overlap between CO_2^- PDOS and total DOS in

Table S1: Fermi levels of OS-ET structures.

OS-ET	2K-CO_2	2K-CO_2 anion	Water-CO_2	Water-CO_2 anion
Fermi levels (eV)	-4.15±0.19	-4.43±0.22	-4.43±0.19	-4.35±0.25

water-CO_2^- system, which is not observed for the water-CO_2 system (Fig. S13 and S14). These observations support the recent arguments[3] that the Fermi level is not the correct quantity for determining the electrode potential in outer-sphere reactions and that the electrode inner potential should instead be used to characterize the electrode potential.

S.1.3. Clarifications on Marcus theory, energy gap, and reaction free energy

Marcus theory can be derived in several but equally valid ways. The linear response approximation is the central assumption behind the famous Marcus barrier equation.

$$\Delta A^{\ddagger}_{Marcus} = \frac{(\Delta A + \lambda)^2}{4\lambda} \tag{S1}$$

$\Delta A^{\ddagger}_{Marcus}$ arises only when the diabatic energy curves are harmonic along the energy gap coordinate: this condition is met when the energy gap distributions are Gaussian, which is equivalent to the linear response approximation as well as the second cumulant expansion.[4, 5] In these cases, the iconic Marcus barrier can be obtained by simulating the initial and final states only. However, in the non-linear Marcus theory, thermodynamic integration at multiple points along the reaction coordinate (energy gap) is required.[6] Studying the non-linear effects would be very interesting, but here we restrict to the linear response theory.

In this work, we follow the cumulant expansion route based on Zwanzig's linear response theory[4, 5], but, as stressed above, this is formally equal to the Gaussian energy gap or harmonic diabatic energy approximations. Within this linear response approximation and cumulant expansion, the reorganization energy can be computed exactly[4, 5] from the energy gap variance: this is equation 4a in Methods. Similarly, equation 4b in Methods arises from cumulant expansion and hence the linear response theory[4, 5], and the reaction free energy is given in terms of the energy gap expectation value and variance through the reorganization energy. Hence, both the reaction energy and reorganization energy depend on the energy gap distributions.

S.1.4. Constrained DFT, electron transfer, and Marcus theory

Constrained DFT (cDFT)[7] is a method for building charge/spin localized or diabatic states with a user-defined charge and/or spin state, being a useful tool for widening the scope of ground-state DFT to excitation processes,

correcting for self-interaction energy in current DFT functionals, excitation energy, and electron transfer (ET) as well as parametrizing model Hamiltonians, for example. cDFT works by specifying an additional constraining term to the KS functional, and the role of this constraint is to enforce a user-specified charge/spin state on the chosen regions of atoms.

For ET during CO_2-to-CO_2^-, two diabatic or charge-localized states are generated using constrained DFT as implemented in the GPAW code.[8] The CO_2 and CO_2^- diabatic states are constructed by forcing the CO_2 molecule to carry either zero or -1 charge, respectively.

Marcus theory has served as the standard theoretical framework for ET reactions for a considerable time. Marcus theory assumes weak coupling between the electronic states of the reactants and products, and assumes a linear response approximation, implying that the surrounding environment of the molecules undergoing ET responds linearly to the event. In this approximation, the free energy profiles of the two ET states can be represented as intersecting parabolas. Two key parameters define the reactant and product free energy curves: the driving force (ΔA), which represents the energy difference between the reactant and product states, and the reorganization energy (λ), which quantifies the energy cost associated with rearranging the reactant or product to reach equilibrium. The Marcus expression for the ET rate is derived from these free energy profiles using classical transition-state theory.

S.2 Enhanced sampling

DFT molecular dynamics (DFT-MD) simulations with slow-growth (SG) sampling approach[9, 10] as implemented in VASP, are performed to evaluate the kinetic barriers of IS-ET for the CO_2-to-$CO_2^{\delta-}$(ads) reaction. In SG-DFT-MD, a suitable collective variable (namely ξ) is chosen as the reaction coordinate and is linearly changed from the characteristic value of the initial state to the final state with a transformation velocity $\dot{\xi}$. The work required to perform the transformation from the initial to the final state can be computed as:

$$\mathrm{W}_{\text{initial-to-final}} = \int_{\xi(\text{initial})}^{\xi(\text{final})} \left(\frac{\partial \mathrm{F}}{\partial \xi}\right) \cdot \dot{\xi} \mathrm{d}t \tag{S2}$$

where F is the time-dependent free energy and $\frac{\partial \mathrm{F}}{\partial \xi}$ can be computed using SG-DFT-MD through the blue-moon ensemble sampling with the SHAKE algorithm.[11] At the limit of infinitesimally small $\partial\xi$, the needed work ($\mathrm{W}_{initial-to-final}$) corresponds to the adiabatic free energy difference between the final and initial states. In the SG sampling, $\partial\xi$=0.001 Å was adopted and applied at each SG-DFT-MD step after testing the shorter step size for the "slow-growth".

The reaction barriers and reaction energies can be obtained by computing the free energy profiles after thermodynamic integration,[11, 12] which was carried out using in-house codes/scripts to post-process the output data to smoothen the data by using a running mean.

S.3 Charge transfer analysis for IS-ET at Au-water interfaces

Bader charge analysis[13, 14] is applied to evaluate the electron transfer between different parts of the system. The analysis results during SG-DFT-MD simulations for IS-ET at Au-water interfaces without any cations, are shown in Fig. S2, indicating that CO_2 would be partially reduced by receiving 0.31 e from Au and water solvents if the inner-sphere reduction could occur.

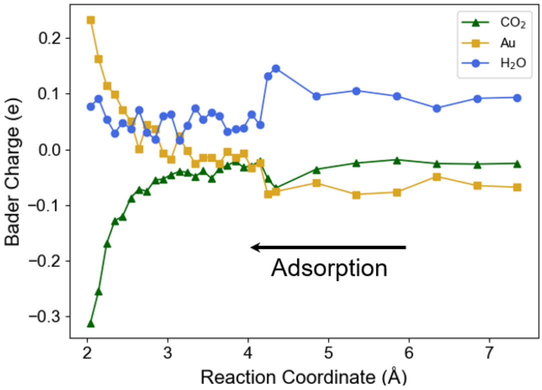

Fig. S2: Bader charge analysis results for IS-ET during SG-DFT-MD at Au-water interfaces.

S.4 Structures and analysis for IS-ET at Au-water-2Li interfaces

The inner-sphere CO_2 activation is simulated at Au-water-2Li interfaces using the SG-DFT-MD methods. Fig. S3 displays the free energy profiles and Li-O (CO_2) distances for the CO_2 adsorption process. The Li^+-O bond distances (Fig. S3b) vary between 2.4 and 4.5 Å, and are on average longer than the bond lengths of 1.98 $\sim$ 2.63 Å for the ionic $LiCO_2$ solid.[15]

S.5 Charge transfer and bonding analysis for CO_2–K^+ interactions

Below we present the charge transfer and bonding analysis for the CO_2 molecule in water and K^+ electrolytes. We show the results for a representative geometry obtained from the middle of the 4ps the AIMD trajectories used in Fig. 3 to study the distance between CO_2 from the Au surface in pure water and the electrolyte solution. We also analyze several other geometries and the

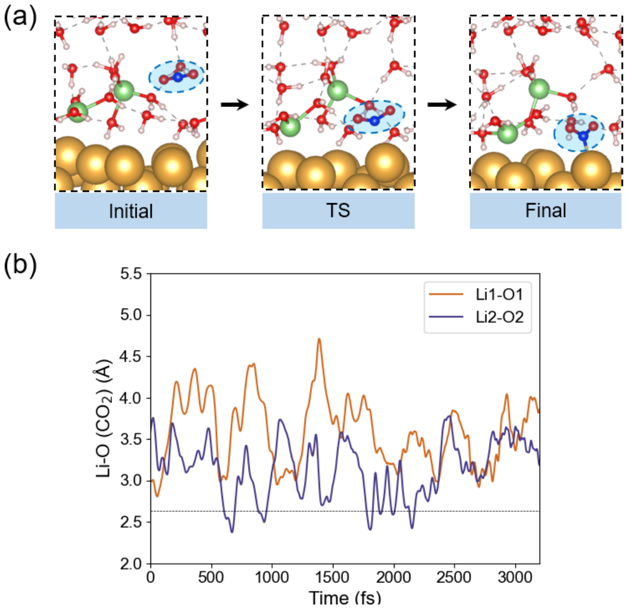

Fig. S3: Key structures (a) and Li-O (CO_2) distance for IS-ET during SG-DFT-MD at Au-water-2Li interfaces.

results are qualitatively and quantitatively similar. These analyses are carried out using GPAW with the setups provided in the computational methods section.

S.5.1. Bonding analysis

To study the interactions between CO_2 and K^+ we turn to the electronic localization function (ELF) which can distinguish between regions of covalent bonds, lone-pairs, metallic bonds, ionic bonds, etc. based on the electronic kinetic energy density.[16–18] In Fig. S4, we present the 2D ELF analysis on the plane connecting the K^+, O, and C in a configuration where CO_2 and K^+ are coordinated. The figure shows that there are no indications of covalent bonding between CO_2 and K. Instead, the lone pair from the oxygen atom is directly pointed towards the cation. This strongly indicates that CO_2 and K^+ interaction is mediated by a short-range Coulombic interaction between the positively charged K^+ ion and oxygen lone pair belonging the oxygen atom carrying a partial negative charge.

S.5.2. Charge transfer

The charge transfer effects between CO_2 and water/electrolyte environments are studied by computing the electron density difference as follows

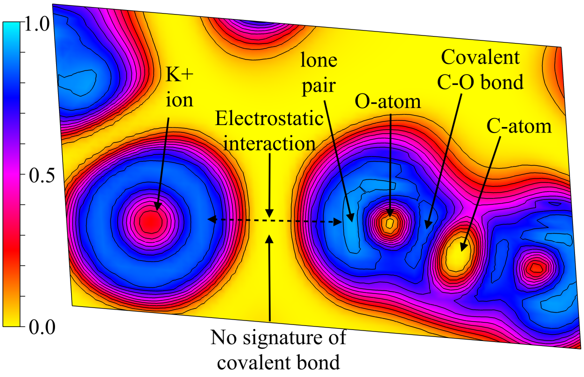

Fig. S4: ELF analysis of the interactions between CO_2 and K^+. Different atoms and bond interactions are indicated. The color scale designates the non-dimensional ELF index where blue areas and values close to unity correspond to areas of high electron localization whereas yellow areas with values around zero denote delocalized electrons or regions of very small electron density.

$$\Delta\rho(\mathbf{r}) = \rho(\mathbf{r})_{CO_2\,at\,interface} - \rho(\mathbf{r})_{CO_2\,in\,vacuum} - \rho(\mathbf{r})_{interface\,without\,CO_2} \quad \text{(S3)}$$

The results in Fig. S5 show that there is minor charge reorganization within individual molecules when CO_2 is brought from the vacuum into the water or electrolyte solution. The charge transfer between different atoms is quantified by applying Bader charge partitioning on $\Delta\rho(\mathbf{r})$ and the results are tabulated in Table S2. This analysis shows that charge transfer between different atoms and molecules in the system is negligibly small and that the minor charge reorganization can be understood as electronic polarization when CO_2 is inserted in water or the K^+ electrolyte.

Table S2: Bader charge analysis on the charge density difference. Positive values indicate electron accumulation, negative values indicate electron depletion.

System	CO_2 in water	CO_2 in K^+ solution
Atom	Δ q	Δ q
K^+	–	-0.000931
O1 in CO_2	0.006031	0.001945
O2 in CO_2	-0.002205	-0.009412
C in CO_2	-0.001669	-0.004768
O atom from H_2O near CO_2	0.007921	0.006020

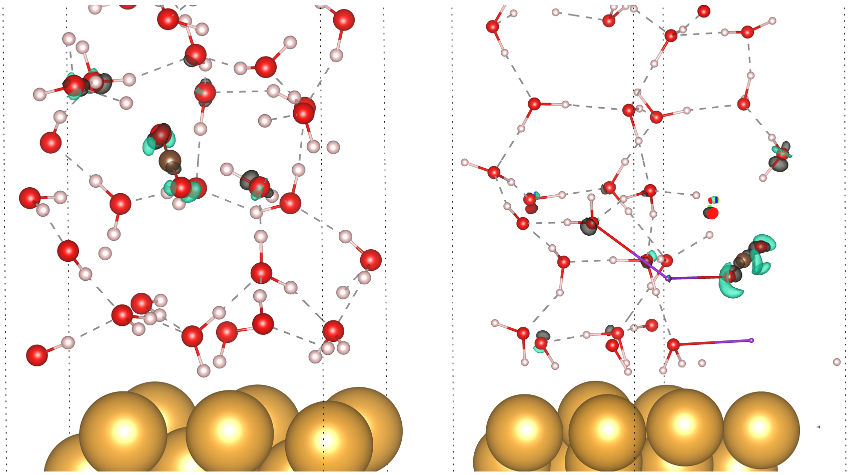

Fig. S5: $\Delta\rho(\mathbf{r})$ plots for CO_2 in water (left) and K^+ solution. The black (turquoise) isosurface corresponds to regions electron accumulation (depletion) at the value 0.0015 e/Å^3.

S.6 AM^+–CO_2 distance analysis during OS-ET

The distance analysis of AM^+–O (CO_2) during OS-ET in cDFT-MD is performed in all of the simulation systems, including CO_2 and CO_2 anion at Au-water-2K interfaces and Au-water-2Li interfaces. For each system, 10 ps cDFT-MD is carried out, and Fig. S6 shows that there is very minor direct coordination between cations and CO_2. It should be noted that the reference values for coordination interaction, e.g., 2.94 Å for K–O and 2.63 Å for Li–O, originate from the crystalline KCO_2 and $LiCO_2$.[15]

S.7 Analysis of MD trajectories

Here we specify the technical details for analyzing the MD trajectories.

S.7.1. Vibrational spectra

The vibrational spectra are computed from velocity-velocity autocorrelation functions as[19]

$$C_{vv}(t) = \sum_i C^i_{vv}(t) = \sum_i \langle v_i(t) \cdot v_i(t=0) \rangle \tag{S4}$$

where i denotes atom i, v_i is the velocity, and $\langle X \rangle$ is the thermodynamic expectation value of X obtained by MD sampling. The vibrational frequencies are then obtained from the spectral density of $C_{vv}(t)$ as

$$I_{vv}(\omega) \propto \int_0^\tau dt C^i_{vv}(t) \cos(\omega t) \tag{S5}$$

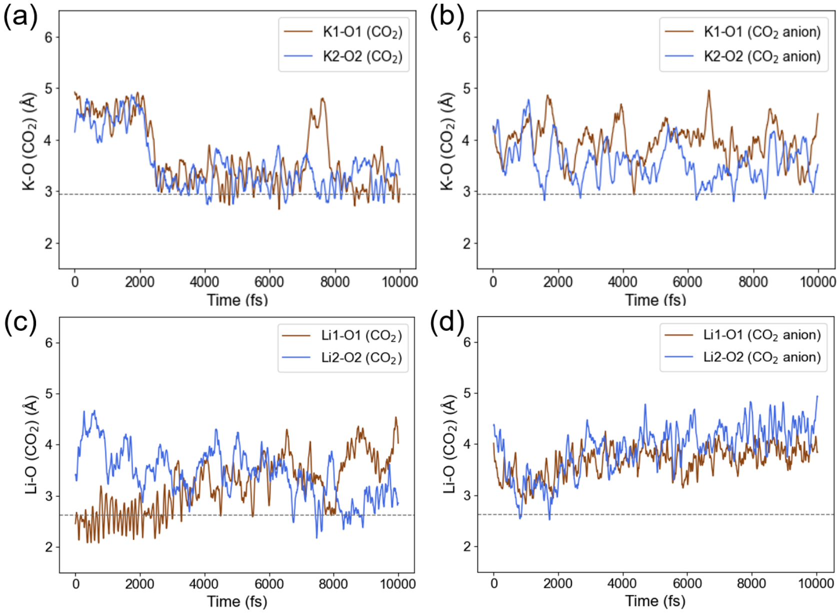

Fig. S6: Distance analysis during cDFT-MD at Au-water-2K interfaces including K-O (CO_2) (a) and K-O (CO_2 anion) (b), and at Au-water-2Li interfaces including Li-O (CO_2) (c) and Li-O (CO_2 anion) (d).

The spectral density I_{vv} contains only the vibrational peak positions and cannot be used for computing absolute (IR or Raman) intensities. As such, I_{vv} contains information about both IR and Raman active modes which is actually beneficial in the present case to enable comparing the simulations with both IR and Raman measurements.

S.7.2. Rotational dynamics

The water rotational dynamics are computed from either the bond direction or dipole direction autocorrelation functions following Ref. 20. The direction-direction autocorrelation function C_{dd} for water molecules is

$$C_{dd}(t) = \sum_{w} C^{w}_{dd}(t) = \sum_{w} \langle P_l \left(d^w(t) \cdot d^w(0) \right) \rangle \qquad \text{(S6)}$$

where P_l is Legendre polynomial of degree l, w denotes a single water molecule, and d^w is any vector joining any points of a water molecule – we used O-H and dipole directions. The different degrees of P_l correspond to different ranks of the rotational dynamics which in turn depend on the experimental technique used for measuring the rotational dynamics. $l = 2$ corresponds to the relaxation of orientational correlation in Raman or magnetic resonance measurements whereas $l = 1$ corresponds to dielectric relaxation times.[21]

As we are interested in electron transfer kinetics where the dynamic solvent corrections or properties are related to dielectric relaxation times[22–24], we have used $l = 1$.

The orientational relaxation time constant, τ_{dd} is computed as a time integral over the corresponding correlation function[20]

$$\tau_{dd} = \int_0^t dt C_{dd}(t) \tag{S7}$$

which be used to characterize the solvent dynamics.

S.7.3. Computation of autocorrelation functions

Correlation functions can be computed as a convolution between quantities at different points in time.[25] The convolution can be conveniently achieved using fast Fourier transforms. This is particularly convenient for autocorrelation functions as the autocorrelation in the Fourier space is just the square modulus of the Fourier transformed time-dependent quantity of interest (here $d^w(t)$ or $v^i(t)$) and the autocorrelation is recovered after an inverse Fourier transform. This procedure is efficiently implemented Numpy[26] in the correlate-function which we employed in all autocorrelation function calculations.

For computing the vibrational spectra, $C_{vv}(t)$ is smoothened using a Blackman-Harris filter as implemented in Scipy.[27] To define individual water molecules, i.e. which hydrogen and oxygen atoms are connected in different water molecules, we used the neighborlist and connected functionalities in ASE.[28]

The used scripts can be obtained from https://gitlab.jyu.fi/mamimela/compel/-/tree/master/scripts.

S.7.4. Orientational dynamics of water

The C_{dd} spectra are shown in Fig. S7 and the calculated orientational relaxation time constants are summarized in Table S3.

Table S3: The calculated orientational relaxation time constants (fs) are summarized, including the CO_2 and CO_2^- in Au-water and Au-water-2K systems with cDFT-MD trajectories.

cDFT-MD trajectory	CO_2	CO_2^-
Au-water	4565	4439
Au-water-2K	4719	4562

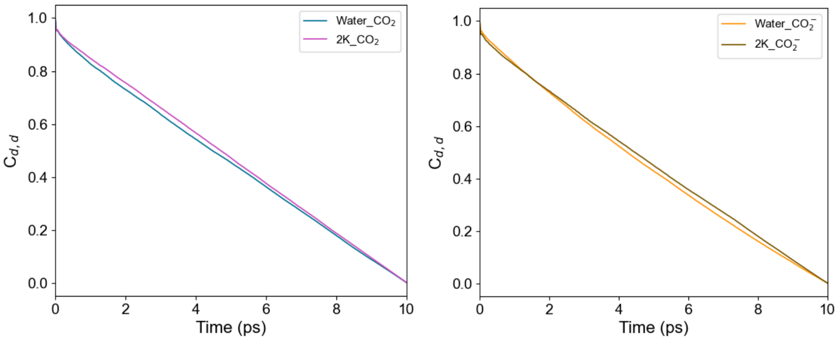

Fig. S7: Time dependence of orientational correlation function C_{dd} for the rotation of dipole vectors of water molecules for CO_2 (left) and CO_2^- (right).

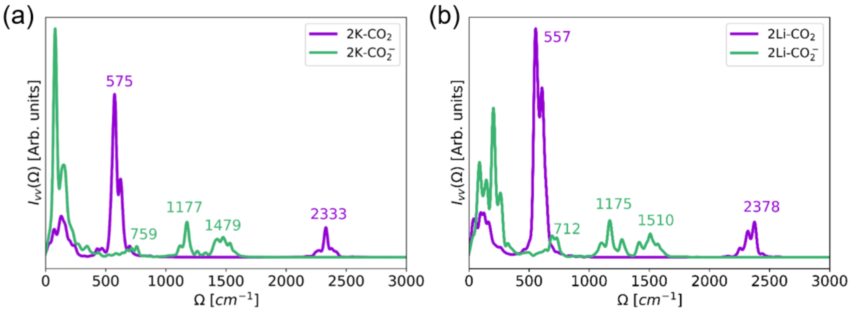

Fig. S8: Simulated vibration spectra of CO_2 and CO_2^- during cDFT-MD for Au-water-2K (a) and Au-water-2Li (b) interfaces.

S.8 Evaluation of electric field effect

Here, to explore the possible electric field effect, we build a large (4 × 3) supercell model with the same number of K^+ cations at Au-water interfaces, thus constructing the weaker average electric field compared to the (2 × 3) supercell used in previous simulations.

S.8.1. Simulation models and parameter comparison

The (2 × 3) and (4 × 3) models are shown in Fig. S9, and lattice parameters are summarized into Table S4. For (2 × 3) and (4 × 3) models, where CO_2 molecules are coordinated by cations, the electric field effect can be qualitatively studied by computing and comparing the reaction barriers via SG-AIMD simulations on IS-ET CO_2 reductions.

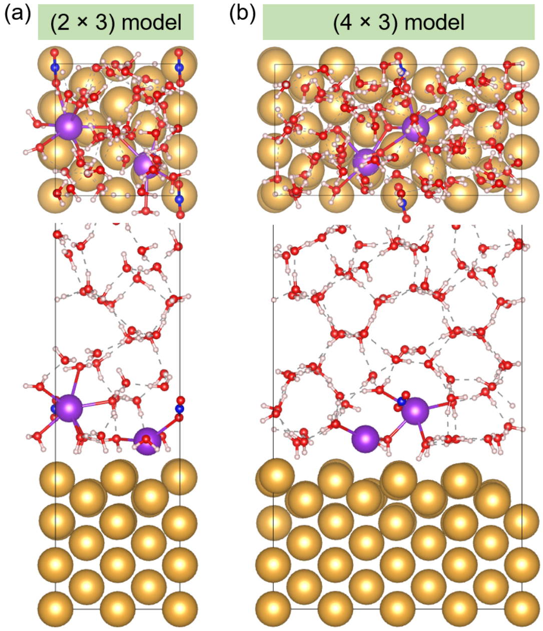

Fig. S9: Top and side view of (2 × 3) model (a) and (4 × 3) model (b).

Table S4: Parameter comparison of (2 × 3) and (4 × 3) simulation models.

Simulation models	Lattice (Å^3)	Total atoms	Water	Cations
(2 × 3) model	8.32 × 8.82 × 40	173	42	2
(4 × 3) model	16.64 × 8.82 × 40	350	87	2

Table S5: The final states of $^*CO_2^{\delta-}$ in (2 × 3) and (4 × 3) simulation models are compared with structure features summarized below.

Simulation models	C-O bond (Å)	O-C-O angle (°)	C-Au (Å)	Charge (e)
(2 × 3) model	1.23&1.25	130	2.12	0.81
(4 × 3) model	1.20&1.21	155	2.44	0.31

S.8.2. SG-AIMD results in (4 × 3) supercell with cation coordination

For the (4 × 3) supercell models with CO_2 coordinated by cations, the reaction barrier for IS-ET is obtained via SG-AIMD simulations on the CO_2 adsorption as shown in Fig. S10. The final states of $^*CO_2^{\delta-}$ in (2 × 3) and (4 × 3) simulation models are compared with structure features summarized into Table S5.

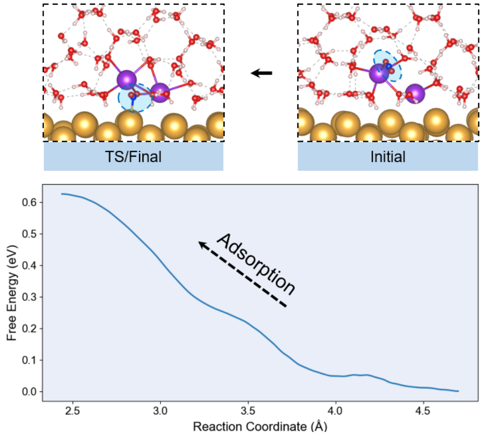

Fig. S10: The key structures (top) and integrated free energy profile (bottom) of CO_2 adsorption in the (4 × 3) supercell model with cation coordination. The maximum evolves quite close to the ending point of the curve.

S.8.3. SG-AIMD results in (4 × 3) supercell without cation coordination

In the (4 × 3) supercell models, we are able to tune the positions of CO_2, which could be either coordinated or non-coordinated by cations, and thus the short-range coordination effect can be differentiated from the electric field by studying the IS-ET kinetics. The basic model for cation-non-coordinated CO_2 in (4 × 3) supercell is shown in Fig. S11, and the free energy profile along with key structures of SG-AIMD is shown in Fig. S12.

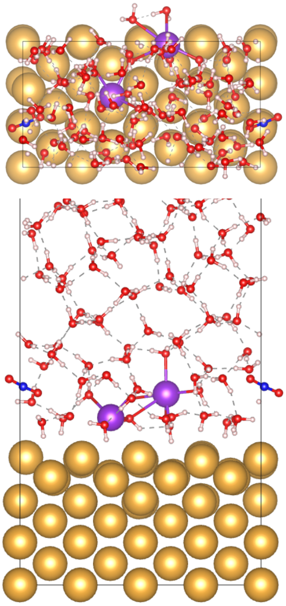

Fig. S11: The (4 × 3) supercell model without cation coordination.

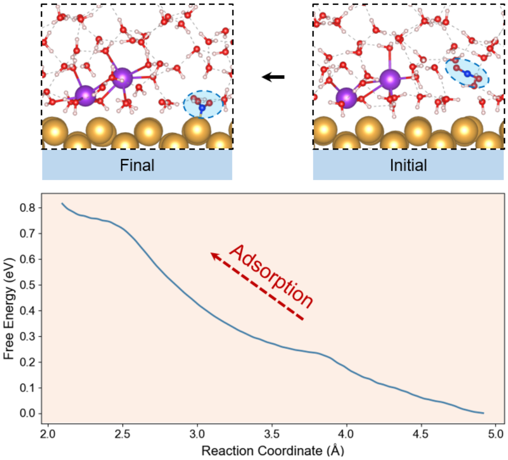

Fig. S12: The key structures (top) and integrated free energy profile (bottom) of CO_2 adsorption in the (4 × 3) supercell model without cation coordination.

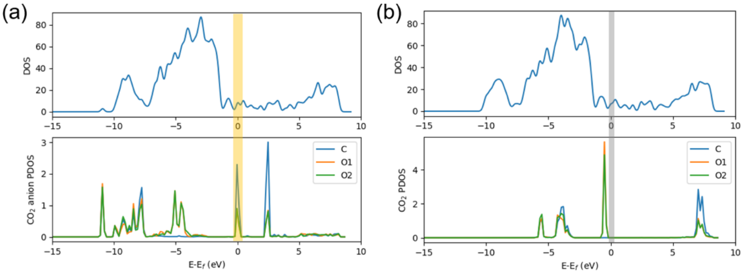

Fig. S13: Density of states (DOS) and projected density of states (PDOS) analysis in water-CO_2^- system (a) and water-CO_2 system (b). Near the Fermi level (E-E$_f$ = 0 eV), the overlap between CO_2^- PDOS and total DOS is marked by yellow in a, and that between CO_2 PDOS and total DOS is marked by gray in b.

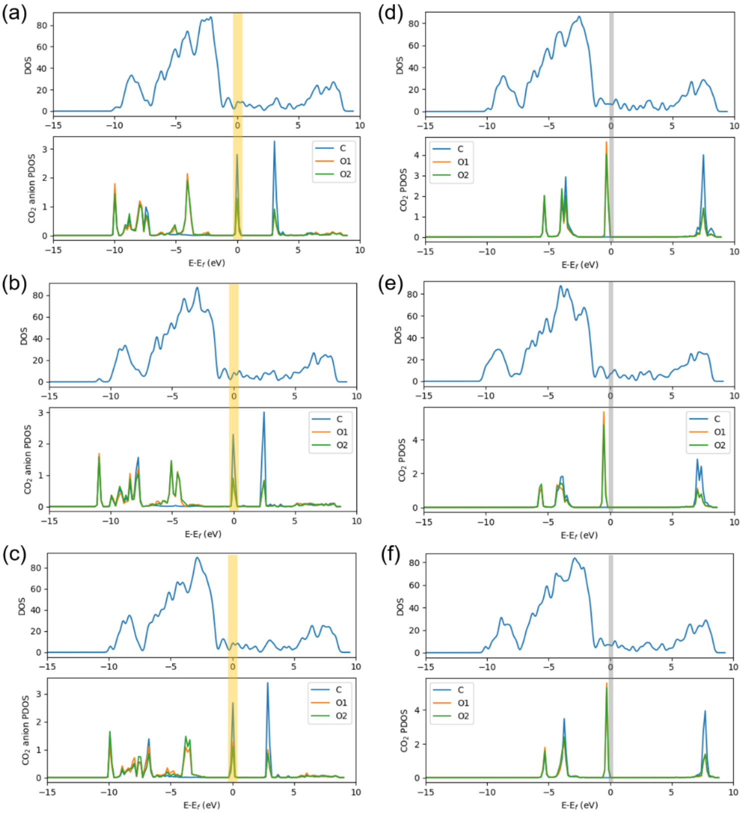

Fig. S14: Density of states (DOS) and projected density of states (PDOS) analysis in water-CO_2^- system (a, b, and c) and water-CO_2 (d, e, and f) with various sampling points during OS-ET simulations.

Supplementary references

[1] Cencer, M. M. *et al.* Interactions of CO_2 anion radicals with electrolyte environments from first-principles simulations. *ACS Omega* **7**, 18131–18138 (2022) .

[2] Chan, K. & Nørskov, J. K. Electrochemical barriers made simple. *J. Phys. Chem. Lett.* **6**, 2663–2668 (2015) .

[3] Melander, M., Wu, T. & Honkala, K. Constant inner potential DFT for modelling electrochemical systems under constant potential and bias (2023) .

[4] Zhou, H. & Szabo, A. Microscopic formulation of Marcus' theory of electron transfer. *J. Chem. Phys.* **103**, 3481–3494 (1995) .

[5] Georgievskii, Y., Hsu, C.-P. & Marcus, R. A. Linear response in theory of electron transfer reactions as an alternative to the molecular harmonic oscillator model. *J. Chem. Phys.* **110**, 5307–5317 (1999) .

[6] Vuilleumier, R., Tay, K. A., Jeanmairet, G., Borgis, D. & Boutin, A. Extension of marcus picture for electron transfer reactions with large solvation changes. *J. Am. Chem. Soc.* **134**, 2067–2074 (2012) .

[7] Kaduk, B., Kowalczyk, T. & Van Voorhis, T. Constrained density functional theory. *Chem. Rev.* **112**, 321–370 (2012) .

[8] Melander, M., Jónsson, E. Ö., Mortensen, J. J., Vegge, T. & García Lastra, J. M. Implementation of constrained DFT for computing charge transfer rates within the projector augmented wave method. *J. Chem. Theory Comput.* **12**, 5367–5378 (2016) .

[9] Woo, T. K., Margl, P. M., Blöchl, P. E. & Ziegler, T. A combined Car-Parrinello QM/MM implementation for ab initio molecular dynamics simulations of extended systems: Application to transition metal catalysis. *J. Phys. Chem. B* **101**, 7877–7880 (1997) .

[10] Jarzynski, C. Nonequilibrium equality for free energy differences. *Phys. Rev. Lett.* **78**, 2690–2693 (1997) .

[11] Sprik, M. & Ciccotti, G. Free energy from constrained molecular dynamics. *J. Chem. Phys.* **109**, 7737–7744 (1998) .

[12] Hassanali, A. A., Cuny, J., Verdolino, V. & Parrinello, M. Aqueous solutions: state of the art in *ab initio* molecular dynamics. *Phil. Trans. R. Soc. A.* **372**, 20120482 (2014) .

[13] Henkelman, G., Arnaldsson, A. & Jónsson, H. A fast and robust algorithm for bader decomposition of charge density. *Comput. Mater. Sci.* **36**, 354–360 (2006) .

[14] Yu, M. & Trinkle, D. R. Accurate and efficient algorithm for bader charge integration. *J. Chem. Phys.* **134**, 064111 (2011) .

[15] Jain, A. *et al.* Commentary: The materials project: A materials genome approach to accelerating materials innovation. *APL Mater.* **1**, 011002 (2013) .

[16] Becke, A. D. & Edgecombe, K. E. A simple measure of electron localization in atomic and molecular systems. *J. Chem. Phys.* **92**, 5397–5403 (1990) .

[17] Savin, A., Nesper, R., Wengert, S. & Fässler, T. F. Elf: The electron localization function. *Angew. Chem. Int. Ed.* **36**, 1808–1832 (1997) .

[18] Koumpouras, K. & Larsson, J. A. Distinguishing between chemical bonding and physical binding using electron localization function (ELF). *J. Phys.: Condens. Matter* **32** (31), 315502 (2020) .

[19] Praprotnik, M. & Janežič, D. Molecular dynamics integration and molecular vibrational theory. ii. simulation of nonlinear molecules. *J. Chem. Phys.* **122**, 174102 (2005) .

[20] Bankura, A., Karmakar, A., Carnevale, V., Chandra, A. & Klein, M. L. Structure, dynamics, and spectral diffusion of water from first-principles molecular dynamics. *J. Phys. Chem. C* **118**, 29401–29411 (2014) .

[21] Banerjee, P. & Bagchi, B. Rotational dynamics of polyatomic ions in aqueous solutions: From continuum model to mode-coupling theory, aided by computer simulations. *J. Chem. Phys.* **148**, 224504 (2018) .

[22] Zusman, L. Outer-sphere electron transfer in polar solvents. *Chem. Phys.* **49**, 295–304 (1980) .

[23] Calef, D. F. & Wolynes, P. G. Classical solvent dynamics and electron transfer. 1. continuum theory. *J. Phys. Chem.* **87**, 3387–3400 (1983) .

[24] He, Z.-D., Chen, Y.-X., Santos, E. & Schmickler, W. The pre-exponential factor in electrochemistry. *Angew. Chem. Int. Ed.* **57**, 7948–7956 (2018) .

[25] Allen, M. P. & Tildesley, D. J. *Computer Simulation of Liquids* (Oxford University Press, New York, 1987).

[26] Harris, C. R. *et al.* Array programming with NumPy. *Nat.* **585**, 357–362 (2020) .

[27] Virtanen, P. *et al.* SciPy 1.0: Fundamental Algorithms for Scientific Computing in Python. *Nat. Methods* **17**, 261–272 (2020) .

[28] Larsen, A. H. *et al.* The atomic simulation environment—a Python library for working with atoms. *J. Phys.: Condens. Matter* **29** (27), 273002 (2017) .
